# Correlation between neutrophil-to-high-density lipoprotein cholesterol ratio (NHR) and adverse prognosis in patients who achieve complete recanalization after thrombectomy for acute large vessel occlusion stroke

**DOI:** 10.3389/fneur.2025.1536535

**Published:** 2025-06-04

**Authors:** Shunchao Ci, Di Li, Feng Wang, Ke Li, Lin Yin

**Affiliations:** ^1^Department of Neurology, The Second Hospital of Dalian Medical University, Dalian, China; ^2^Department of Neurological Intervention and Neurological Intensive Care, Central Hospital of Dalian University of Technology, Dalian, China; ^3^Department of Interventional Therapy, The First Hospital of Dalian Medical University, Dalian, China

**Keywords:** neutrophil-to-high-density lipoprotein cholesterol ratio, adverse prognosis, complete recanalization, mechanical thrombectomy, large vessel occlusion

## Abstract

**Background:**

The neutrophil-to-high-density lipoprotein cholesterol ratio (NHR) has emerged as a novel inflammatory marker with prognostic significance. This study aims to explore the association between NHR and adverse prognosis in patients with acute large vessel occlusion (LVO) stroke who achieved complete recanalization after mechanical thrombectomy (MT).

**Methods:**

This retrospective study analyzed acute ischemic stroke (AIS) patients with LVO who underwent MT at three stroke centers in Dalian, China, between January 2016 and November 2023. Complete recanalization was defined as achieving a modified Thrombolysis in Cerebral Infarction (mTICI) grade 3. Blood parameters were assessed within 24 h after MT. We compared intergroup differences based on NHR tertiles and employed the multivariate logistic regression analysis to assess the relationship between NHR and adverse outcomes.

**Results:**

This study included 348 AIS patients with LVO, of whom 215 (61.8%) had adverse clinical outcomes at 90 days. The multivariate logistic regression analysis revealed a significant association between an elevated NHR and 90-day adverse outcomes (OR 2.311, 95% CI 1.248–4.278, *p* = 0.008). A restricted cubic spline curve demonstrated a linear dose–response relationship between NHR and adverse outcomes, with a *p*-value of 0.348 for non-linearity.

**Conclusion:**

Our findings revealed that an elevated NHR could increase the risk of adverse prognosis following complete recanalization after MT in acute LVO stroke patients, which indicated that NHR could serve as a potential inflammatory marker for identifying high risk patients.

## Introduction

Mechanical thrombectomy (MT) has been established as an effective treatment for acute ischemic stroke (AIS) caused by large vessel occlusion (LVO) (1). Successful recanalization is typically defined as achieving modified Thrombolysis in Cerebral Infarction (mTICI) grade 2b-3 (2). Previous studies have demonstrated that successful recanalization can be achieved in over 83% of patients after MT (3, 4). Despite successful recanalization, nearly half of the patients still exhibit unfavorable clinical outcomes at 90 days, a phenomenon termed futile recanalization (5–7). Current studies on futile recanalization primarily focus on patients who achieve successful recanalization (mTICI 2b-3) after MT; however, the understanding of potential factors associated with poor outcomes in patients who achieve complete recanalization (mTICI 3) remains limited. A systematic review and meta-analysis indicated that mTICI 3 is associated with better outcomes and safety compared to mTICI 2b, and the recanalization grade represents the most important modifiable predictor of patient prognosis (8). VanHorn et al. found that patients with complete recanalization still had a high proportion of adverse outcomes (9). Therefore, actively exploring the factors associated with futile recanalization in patients with mTICI grade 3 may help optimize patient management and improve clinical outcomes.

The inflammatory immune response plays a crucial role in the pathophysiology, treatment outcomes, and prognosis of ischemic stroke (10). Among leukocytes, neutrophils are the first immune cells to increase in circulation shortly after the onset of ischemic stroke (11). Neutrophils in the blood infiltrate ischemic or infarcted tissue through the compromised blood–brain barrier (BBB) and release inflammatory mediators, thereby increasing the risk of BBB disruption, reperfusion injury, hemorrhagic transformation, and malignant brain edema (12). High-density lipoprotein cholesterol (HDL-C) regulates macrophages and adipocytes via cholesterol transporters, exerting anti-inflammatory (13) and anti-atherosclerosis effects (14). As a potential inflammatory marker, the neutrophil-to-high-density lipoprotein cholesterol ratio (NHR) has demonstrated certain prognostic value. This blood indicator is inexpensive and readily available. Huang et al. found that an elevated NHR is a potential predictor of long-term mortality and recurrence rates in elderly patients with acute myocardial infarction (15). A previous study indicated that NHR may be associated with an increased risk of adverse short-term outcomes following intravenous thrombolysis in AIS patients (16). However, its correlation with futile recanalization at mTICI3 remains unclear. This study aimed to investigate the relationship between NHR and adverse prognosis in LVO patients who achieved complete recanalization after MT.

## Methods

This multicenter retrospective study consecutively enrolled patients with AIS who underwent MT at three stroke centers: the Second Hospital of Dalian Medical University, the First Hospital of Dalian Medical University, and the Central Hospital of Dalian University of Technology, between January 2016 and November 2023. The inclusion criteria were as follows: (1) age ≥18 years, (2) received MT within 24 h and achieved complete recanalization with mTICI grade 3, and (3) occlusion of the internal carotid artery or M1/M2 segments of the middle cerebral artery. The exclusion criteria were as follows: (1) pre-onset modified Rankin Scale (mRS) score >2; (2) severe infection, malignant tumor, or immune dysfunction; (3) severe cardiac, hepatic, renal, or other major organ diseases; (4) missing medical records or data; and (5) 90-day loss to follow-up. This study was approved by the Ethics Committee of the Second Affiliated Hospital of Dalian Medical University.

Data collection included patient demographics and clinical characteristics such as age, sex, medical history (including hypertension, diabetes, hyperlipidemia, and atrial fibrillation), smoking status, systolic and diastolic blood pressure, National Institutes of Health Stroke Scale (NIHSS) score, Alberta Stroke Program Early CT Score (ASPECTS), intravenous thrombolysis, stroke subtype according to the Trial of Org 10,172 in Acute Stroke Treatment (TOAST) criteria, anesthesia type, retriever passes, time from onset to puncture (OTP), and time from onset to recanalization (OTR). Laboratory tests conducted within 24 h after MT measured neutrophil, lymphocyte, and platelet counts, as well as cholesterol, triglycerides, low-density lipoprotein cholesterol (LDL-C), HDL-C, and NHR. ASPECTS was evaluated using non-contrast computed tomography (NCCT). The site of vascular occlusion was confirmed using digital subtraction angiography (DSA). All neuroimaging data were independently reviewed by two experienced neurointerventionists who were blinded to the patients’ clinical information, and discrepancies were resolved by consensus. Recanalization was assessed using the mTICI score, with complete recanalization defined as an mTICI score of 3 (17). Each center followed up with postoperative patients via telephone or outpatient clinic visits to assess the 90-day mRS score. An mRS score of 0–2 was defined as a good outcome, while a score of 3–6 was considered a poor outcome.

### Statistical analysis

**Figure 1:**
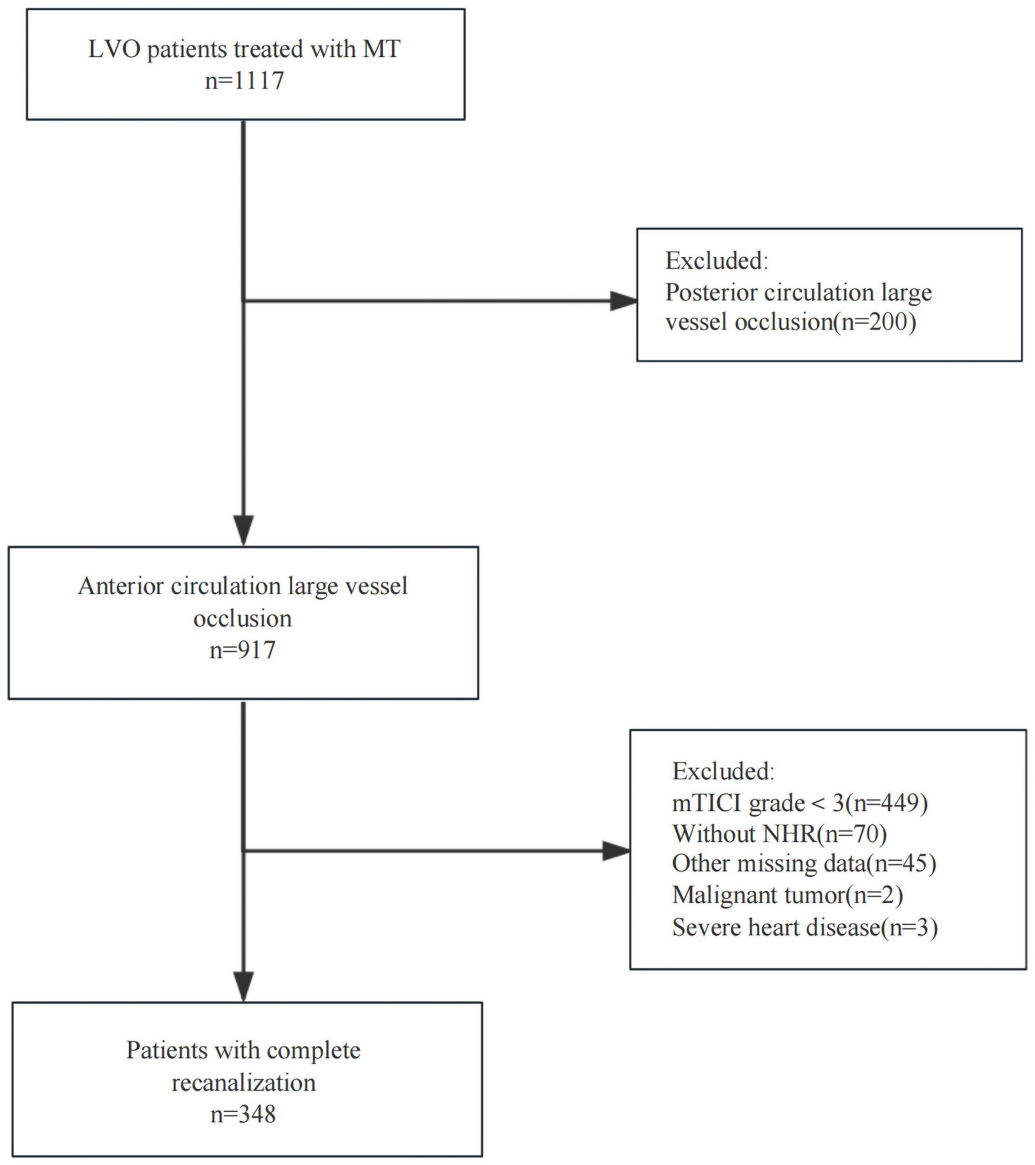
Flowchart showing the number (*n*) of patients included in the analysis. LVO, large vessel occlusion; MT, mechanical thrombectomy; mTICI, modified Thrombolysis in Cerebral Infarction; NHR, neutrophil-to-high-density lipoprotein cholesterol ratio.

Continuous variables were presented as means with standard deviations or medians with interquartile ranges (IQRs). The normality of the distribution was assessed using the Kolmogorov–Smirnov test. Categorical variables were reported as frequencies and percentages. Group differences in continuous variables were evaluated using the Wilcoxon test, whereas the chi-squared or Fisher’s exact test was used for categorical variables. The study population was stratified into tertiles based on the NHR range, and continuous variables were analyzed using a one-way analysis of variance (ANOVA) or the Kruskal-Wallis test. Confounding factors included in the multivariate logistic regression models were those that showed statistical significance in the univariate analysis. Model 1 remained unadjusted, Model 2 was adjusted for demographic factors, and Model 3 accounted for clinically relevant factors. Statistical analyses were performed using SPSS Version 27.0 software (IBM Corp., Armonk, NY, USA) and R version 4.2.3 (R Core Team, Vienna, Austria). A *p*-value of <0.05 was considered statistically significant (Figure 1).

## Results

**Figure 2:**
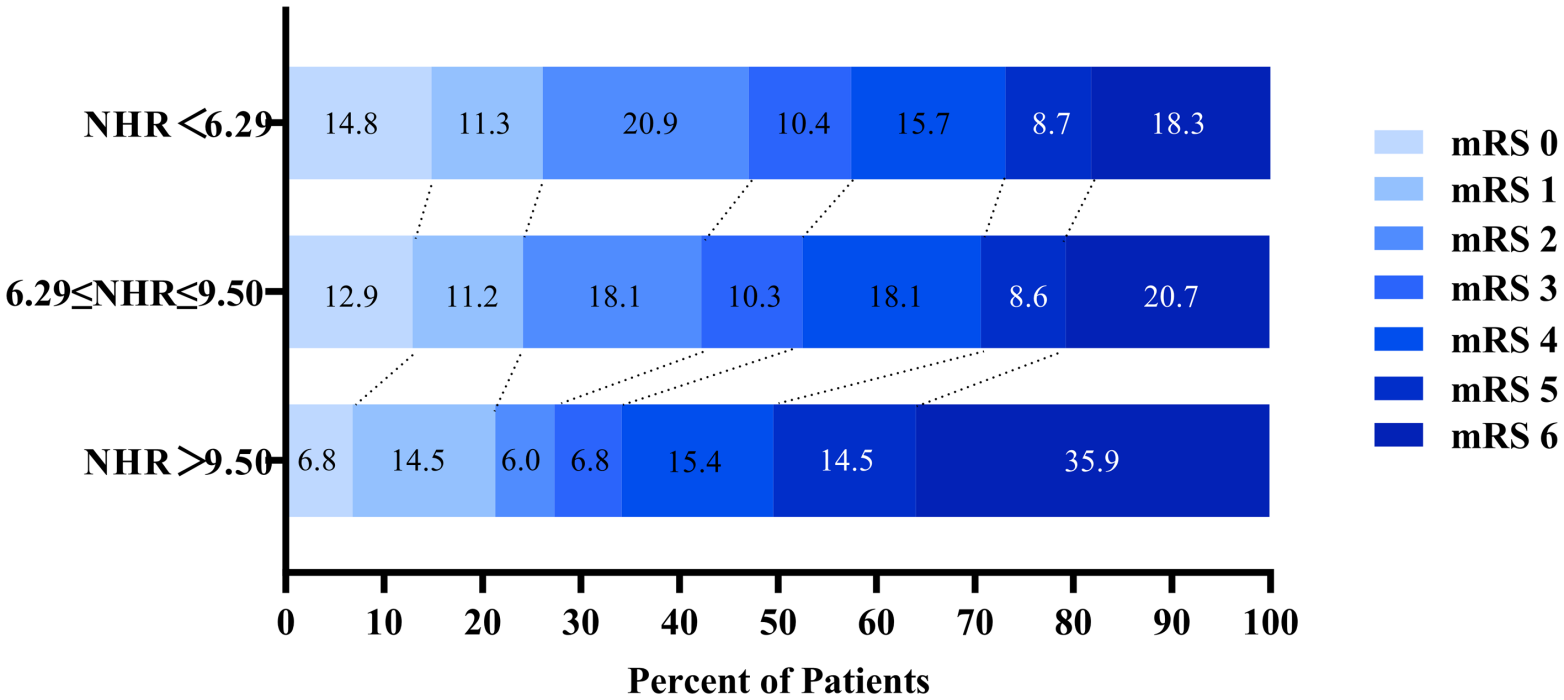
Distribution of modified Rankin Scale (mRS) scores at 90 days stratified by NHR tertiles.

**Figure 3:**
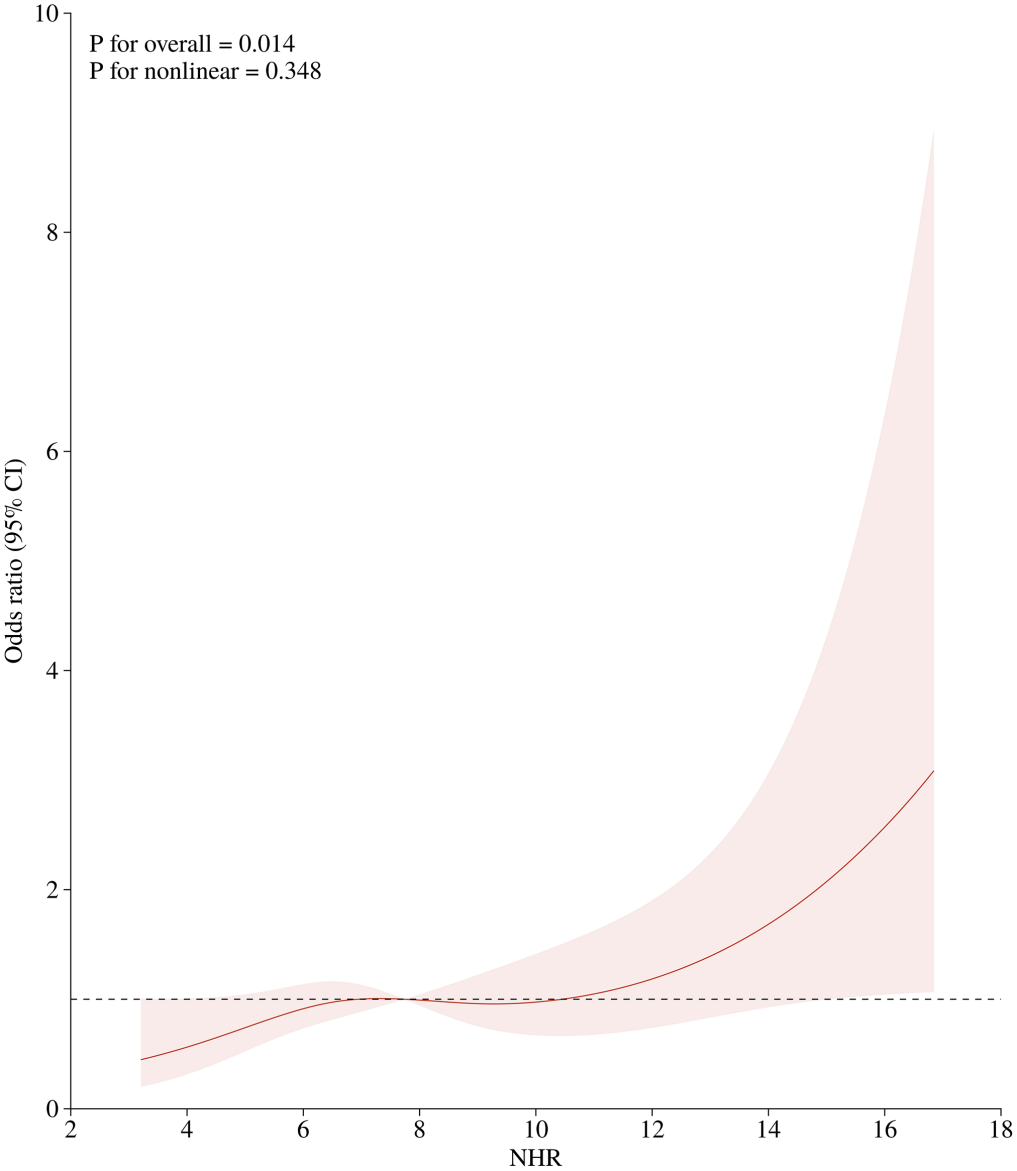
The positive linear relationship between NHR and adverse prognosis 90 days after RCS analysis.

**Figure 4:**
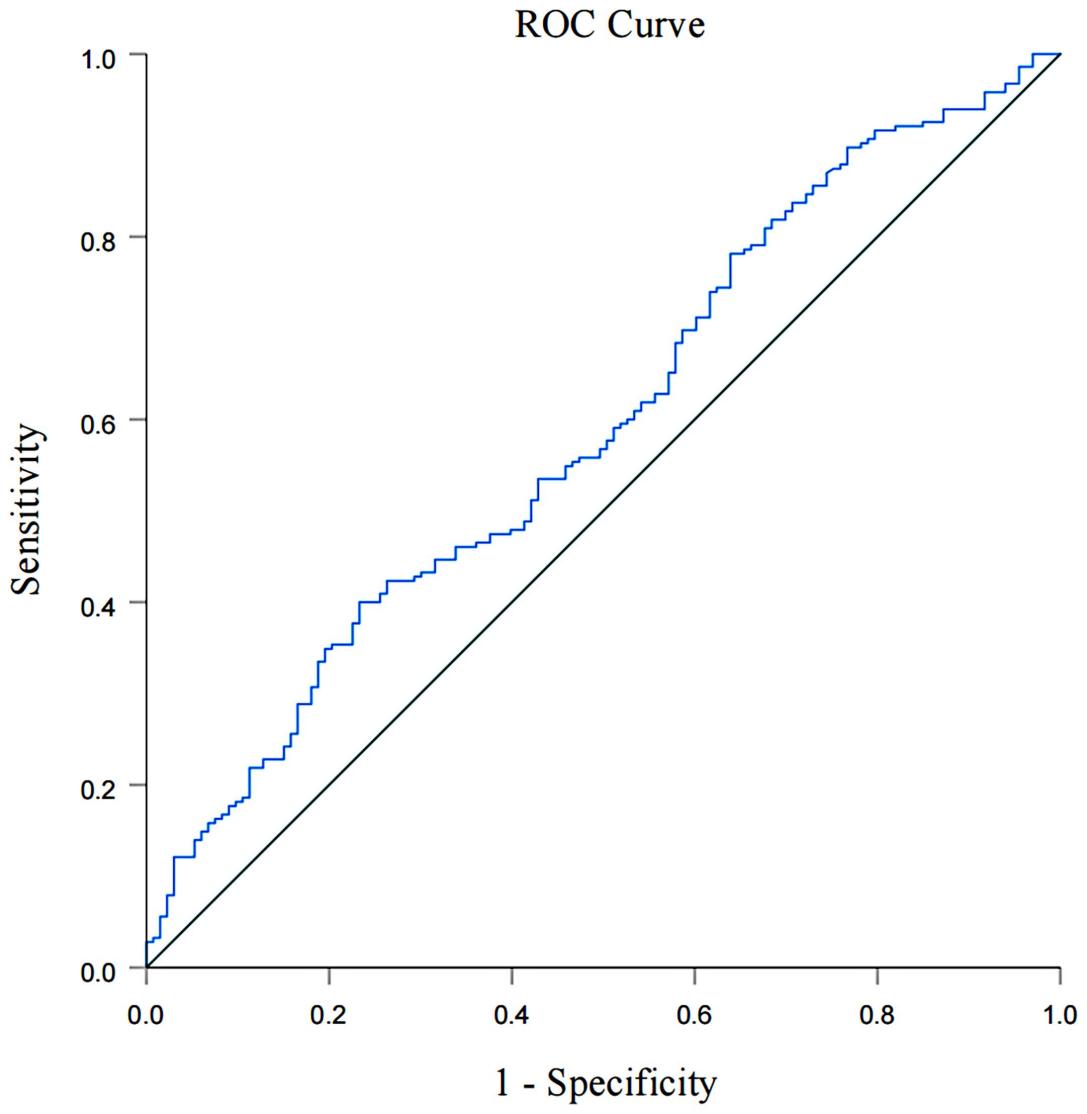
ROC curve analysis of NHR in predicting poor prognosis at 90 days.

**Figure 5:**
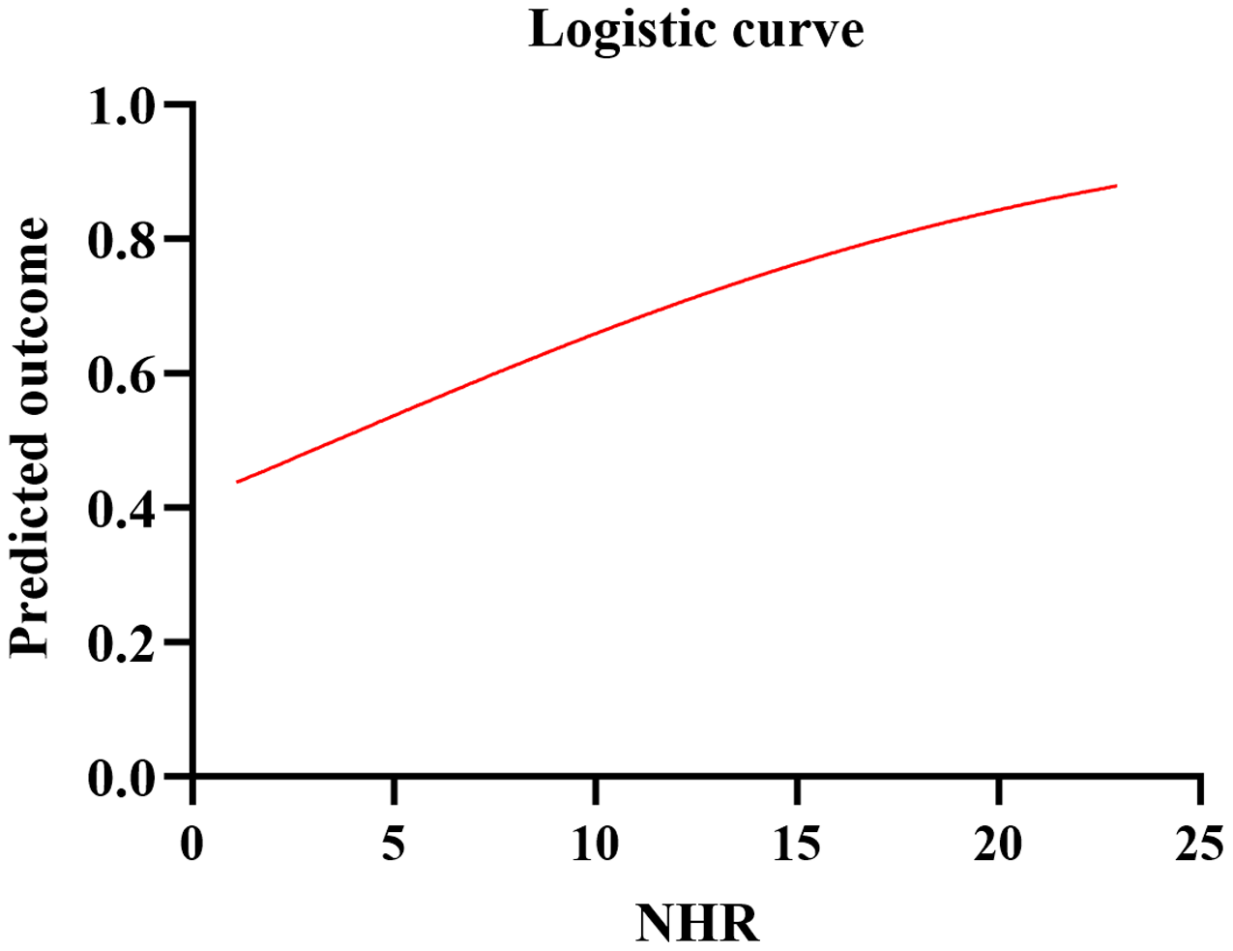
Logistic curve of the predicted probability of poor prognosis and NHR.

**Figure 6:**
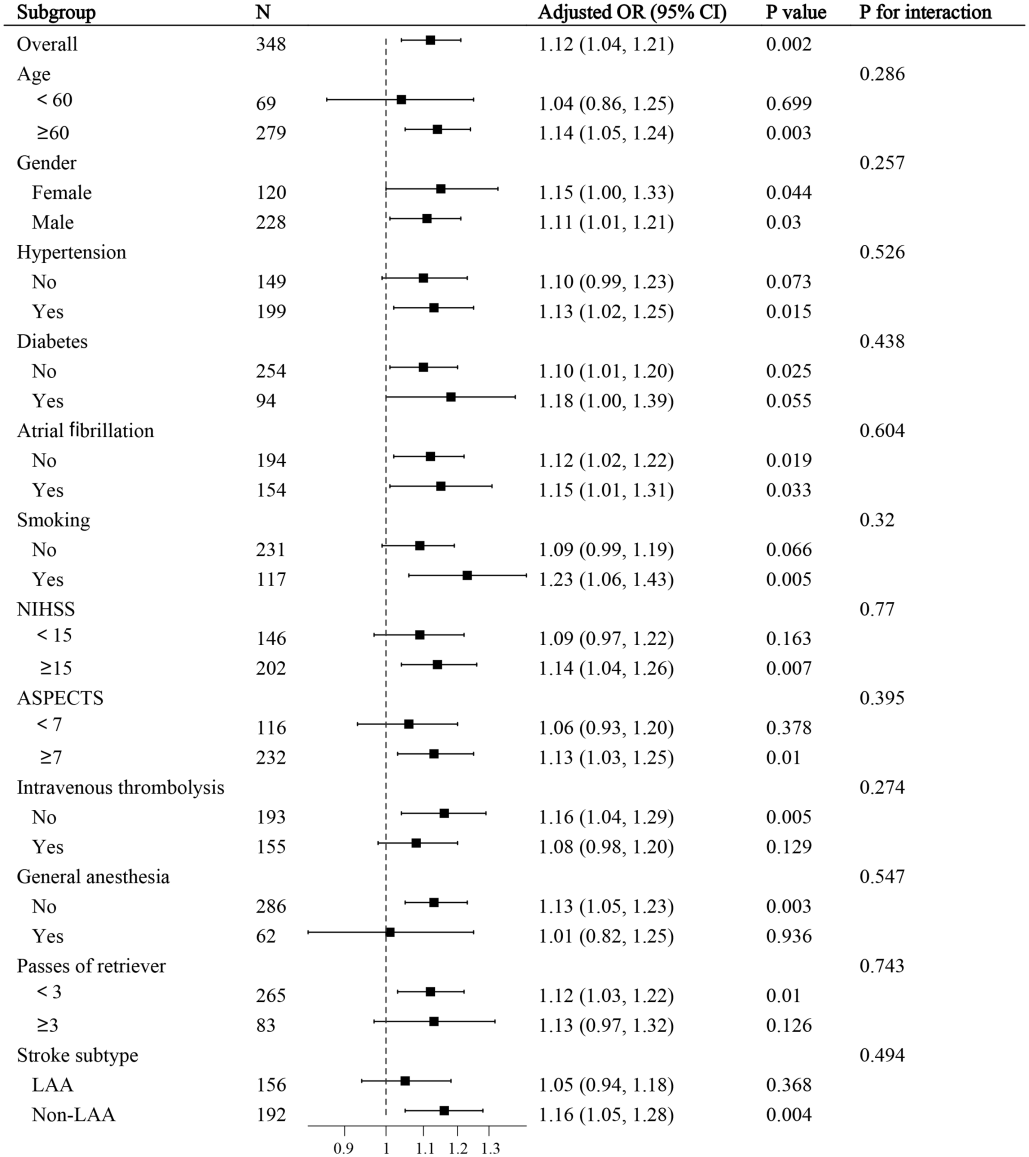
Stratified analysis of the relationship between the NHR and adverse prognosis at 90 days. The model was adjusted for age, sex, hypertension, diabetes, atrial fibrillation, smoking, intravenous thrombolysis, number of passes, baseline NIHSS score, baseline ASPECTS, and stroke subtype. In each subgroup analysis, separate stratified variables were not included.

A total of 917 patients with anterior circulation and LVO who underwent MT were included in this study, of whom 348 met the eligibility criteria. The median age was 70 years (IQR, 62–76), with 228 (65.5%) being male and 215 (61.8%) experiencing adverse clinical outcomes at 90 days. Patients were stratified into three NHR tertiles: first (<6.29), second (6.29–9.50), and third (>9.50). Higher NHR tertiles were associated with a higher proportion of male participants (*p* < 0.001), hyperlipidemia (*p* < 0.001), poor 90-day prognosis (*p* < 0.005), elevated platelet counts (*p* = 0.014), elevated neutrophil counts (*p* < 0.001), elevated TG levels (*p* < 0.001), and lower HDL-C levels (*p* < 0.001) (Table 1). Ordered logistic regression analysis demonstrated significant differences in mRS scores across NHR tertiles, with an adjusted OR of 0.4897 (95% CI: 0.3021–0.7945, *p* = 0.004) between the lowest and highest tertiles (Figure 2). To explore the association between NHR and prognosis, patients were divided into good and adverse prognosis groups for the univariate analysis. Patients with adverse prognoses tended to be older (*p* < 0.001), had a higher prevalence of diabetes mellitus (*p* = 0.014), had elevated baseline NIHSS scores (*p* < 0.001), had reduced ASPECTS scores (*p* < 0.001), had higher baseline systolic blood pressure (*p* = 0.026), and had a higher incidence of general anesthesia use (*p* = 0.012). Laboratory findings indicated that these patients also had higher neutrophil counts (*p* < 0.001), lower lymphocyte counts (*p* = 0.001), and elevated NHR levels (*p* = 0.004) (Table 2). Three models were developed for the multivariate logistic regression analysis. Model 1, which was unadjusted, revealed that the highest NHR group was significantly associated with an increased risk of adverse prognosis after thrombectomy (OR 2.371, 95% CI: 1.368–4.112, *p* = 0.002). Model 2, which was adjusted for demographic factors such as age, demonstrated that NHR remained a significant risk factor for adverse prognosis (OR 2.887, 95% CI: 1.618–5.151, *p* < 0.001). Model 3, further adjusted for clinically relevant confounders (diabetes, general anesthesia, baseline NIHSS, ASPECTS, baseline systolic blood pressure, and lymphocyte count) based on Model 2, confirmed the persistence of this association (OR 2.311, 95% CI: 1.248–4.278, *p* = 0.008). When analyzed as a continuous variable, NHR yielded consistent results (OR 1.445 per 1-SD increase, 95% CI: 1.095–1.908, *p* = 0.009) (Table 3). A restricted cubic spline curve analysis demonstrated a positive linear dose–response relationship between the NHR and adverse prognosis (A *p*-value of 0.348 for non-linearity) (Figure 3). A receiver operating characteristic (ROC) curve was plotted to evaluate the predictive performance of NHR. The results demonstrated that the area under the curve (AUC) was 0.592 (95% CI: 0.531–0.653; *p* = 0.004), with an optimal cutoff value of 9.486 (Figure 4). The logistic curve demonstrated that the probability of adverse prognosis gradually increased with elevated NHR levels (Figure 5). Subgroup analyses revealed that factors such as age, sex, hypertension, diabetes, atrial fibrillation, smoking, intravenous thrombolysis, number of passes, baseline NIHSS, baseline ASPECTS, and stroke subtyping did not significantly influence the correlation between NHR and adverse prognosis (all *p*-values for interaction >0.05) (Figure 6).

**Table 1:** Demographic and clinical characteristics based on NHR tertiles.

Variable	Total (*n* = 348)	NHR<6.29 (*n* = 115)	6.29 ≤ NHR≤9.50 (*n* = 116)	NHR>9.50 (*n* = 117)	*p*-value
Age, median (IQR)	70.0 (62.0–76.0)	71.0 (63.8–77.3)	68.0 (60.0–75.0)	70.0 (61.0–76.0)	0.053
Sex, *n* (%)					<0.001
Male	228 (65.5)	60 (52.2)	79 (68.1)	89 (76.1)	
Female	120 (34.5)	55 (45.8)	37 (30.8)	28 (23.3)	
HTN, *n* (%)	199 (57.2)	74 (66.1)	56 (46.6)	69 (59.0)	0.010
DM, *n* (%)	94 (27.0)	31 (27.0)	28 (24.1)	35 (29.9)	0.611
Dyslipidemia, *n* (%)	155 (44.5)	30 (26.1)	47 (40.5)	78 (66.7)	<0.001
AF, *n* (%)	154 (44.3)	59 (51.3)	50 (43.1)	45 (38.5)	0.137
Smoking, *n* (%)	117 (33.6)	36 (31.3)	31 (26.7)	50 (42.7)	0.029
Intravenous thrombolysis, *n* (%)	155 (44.5)	55 (47.8)	58 (50.0)	42 (35.9)	0.066
NIHSS, median (IQR)	16 (13–20)	16 (13–20)	16 (12–20)	18 (14–22)	0.015
ASPECTS, median (IQR)	8 (7–10)	9 (7–10)	9 (8–10)	8 (6–9)	0.004
Baseline SBP, median (IQR)	146 (130–165)	144 (130–165)	143 (123–160)	151 (133–169)	0.034
Baseline DBP, median (IQR)	81 (73–90)	82 (72–89)	80 (70–90)	85 (75–96)	0.059
OTP, median (IQR)	245.0 (185.3–340.0)	235.0 (170.8–317.5)	270.0 (205.0–350.0)	246.0 (195.0–344.0)	0.031
OTR, median (IQR)	325.0 (254.5–420.0)	291.0 (231.5–400.1)	345.0 (265.0–438.0)	330.0 (267.0–440.0)	0.011
Occlusion site, *n* (%)					0.883
ICA	122 (35.1)	39 (33.9)	41 (35.3)	42 (35.9)	
MCA-M1	161 (46.3)	56 (48.7)	54 (46.6)	51 (43.6)	
MCA-M2	31 (10.1)	13 (11.3)	11 (9.5)	11 (9.4)	
Tandem	30 (8.6)	7 (6.1)	10 (8.6)	13 (11.1)	
TOAST, *n* (%)					0.888
LAA	165 (44.8)	51 (44.3)	45 (38.8)	60 (51.3)	
Cardioembolic	183 (52.6)	61 (53.0)	69 (59.5)	53 (45.3)	
Others	9 (2.6)	3 (2.6)	2 (3.7)	4 (1.4)	
Neutrophils, 10^9^/L, median (IQR)	8.47 (6.58–10.34)	6.15 (5.05–7.31)	8.54 (7.10–9.75)	11.02 (9.17–13.53)	<0.001
Lymphocytes, 10^9^/L, median (IQR)	1.14 (0.80–1.54)	1.07 (0.76–1.51)	1.20 (0.90–1.60)	1.11 (0.80–1.51)	0.179
Platelets, 10^9^/L, median (IQR)	186 (150–224)	176 (140–214)	188 (160–228)	191 (161–232)	0.014
TC, mmol/L, median (IQR)	4.30 (3.66–5.05)	4.23 (3.72–5.16)	4.41 (3.82–5.23)	4.16 (3.51–4.81)	0.067
TG, mmol/L, median (IQR)	1.13 (0.81–1.51)	0.94 (0.69–1.25)	1.16 (0.92–1.53)	1.24 (0.91–1.65)	<0.001
HDL-C, mmol/L, median (IQR)	1.08 (0.90–1.27)	1.27 (1.10–1.49)	1.08 (0.90–1.25)	0.93 (0.77–1.06)	<0.001
LDL-C, mmol/L, median (IQR)	2.60 (2.05–3.28)	2.40 (1.95–3.22)	2.79 (2.12–3.45)	2.52 (2.03–3.22)	0.065
General anesthesia, *n* (%)	62 (17.8)	17 (14.8)	14 (12.1)	31 (26.5)	0.009
Passes of retriever ≥3, *n* (%)	83 (23.9)	25 (21.7)	33 (28.4)	25 (21.4)	0.362
90-day mRS 3–6, *n* (%)	215 (61.8)	62 (53.9)	67 (57.8)	86 (73.5)	0.005

HTN, hypertension; DM, diabetes mellitus; AF, atrial fibrillation; NIHSS, National Institute of Health Stroke Scale; ICA, internal carotid artery; MCA, middle cerebral artery.

TOAST, Trial of Org 10,172 in Acute Stroke Treatment classification; LAA, large artery atherosclerosis; OTP, time from onset to puncture; OTR, time from onset to recanalization.

IQR, interquartile range.

**Table 2:** Comparison of demographics and clinical characteristics between poor and good outcome groups.

Variable	Total (*n* = 348)	Poor outcome (mRS3-6) (*n* = 215)	Good outcome (mRS0-2) (*n* = 133)	*p*-value
Age, median (IQR)	70.0 (62.0–76.0)	72.0 (64.0–78.0)	66.0 (58.5–72.5)	<0.001
Gender, *n* (%)				0.507
Male	228 (65.5)	138 (64.2)	90 (67.7)	
Female	120 (34.5)	77 (35.8)	43 (32.3)	
HTN, *n* (%)	199 (57.2)	129 (60.0)	70 (52.9)	0.177
DM, *n* (%)	94 (27.0)	68 (31.6)	26 (19.5)	0.014
Dyslipidemia, *n* (%)	155 (44.5)	102 (47.4)	53 (39.8)	0.166
AF, *n* (%)	154 (44.3)	102 (47.4)	52 (39.1)	0.128
Smoking, *n* (%)	117 (33.6)	66 (30.7)	51 (38.3)	0.142
Intravenous thrombolysis, *n* (%)	155 (44.5)	94 (43.7)	61 (45.9)	0.696
NIHSS, median (IQR)	16 (13–20)	18 (14–22)	16 (12–18)	<0.001
ASPECTS, median (IQR)	8 (7–10)	8 (6–9)	9 (7–10)	<0.001
Baseline SBP, median (IQR)	146 (130–165)	150 (132–166)	141 (125–160)	0.026
Baseline DBP, median (IQR)	81 (73–90)	82 (75–90)	80 (70–92)	0.398
OTP, median (IQR)	245.0 (185.3–340.0)	247.0 (195.0–340.0)	240.0 (172.5–341.5)	0.447
OTR, median (IQR)	325.0 (254.5–420)	330.0 (260.0–428.0)	305.0 (239.0–413.0)	0.158
Occlusion site, *n* (%)				0.740
ICA	122 (35.1)	81 (37.7)	41 (30.8)	
MCA-M1	161 (46.3)	88 (40.9)	73 (54.9)	
MCA-M2	31 (10.1)	24 (11.2)	11 (8.3)	
Tandem	30 (8.6)	22 (10.2)	8 (6.0)	
TOAST, *n* (%)				0.436
LAA	165 (44.8)	94 (43.7)	62 (46.6)	
Cardioembolic	183 (52.6)	117 (54.4)	66 (49.6)	
Others	9 (2.6)	4 (1.9)	5 (3.8)	
Neutrophils, 10^9^/L, median (IQR)	8.47 (6.58–10.34)	8.82 (7.08–11.18)	7.58 (5.95–9.50)	<0.001
Lymphocytes,10^9^/L, median (IQR)	1.14 (0.80–1.54)	1.07 (0.73–1.45)	1.28 (0.96–1.69)	0.001
Platelets, 109/L, median (IQR)	186 (150–223)	183 (148–217)	195 (155–235)	0.054
TC, mmol/L, median (IQR)	4.30 (3.66–5.05)	4.32 (3.66–5.07)	4.30 (3.64–5.06)	0.780
TG, mmol/L, median (IQR)	1.13 (0.81–1.51)	1.08 (0.78–1.50)	1.15 (0.86–1.58)	0.751
HDL-C, mmol/L, median (IQR)	1.08 (0.90–1.27)	1.10 (0.92–1.29)	1.06 (0.89–1.26)	0.221
LDL-C, mmol/L, median (IQR)	2.60 (2.05–3.28)	2.59 (2.01–3.25)	2.63 (2.08–3.32)	0.348
NHR, median (IQR)	7.74 (5.70–10.53)	7.92 (6.09–11.33)	7.23 (5.10–9.43)	0.004
General anesthesia, *n* (%)	62 (17.8)	47 (21.9)	15 (11.3)	0.012
Passes of retriever ≥3, *n* (%)	83 (23.9)	50 (23.3)	33 (24.8)	0.741

HTN, hypertension; DM, diabetes mellitus; AF, atrial fibrillation; NIHSS, National Institute of Health Stroke Scale; ICA, internal carotid artery; MCA, middle cerebral artery.

TOAST, the Trial of Org 10,172 in Acute Stroke Treatment classification; LAA, large artery atherosclerosis; OTP, time from onset to puncture; OTR, time from onset to recanalization.

NHR, neutrophil-high-density lipoprotein ratio; IQR, interquartile range.

**Table 3:** Multivariable logistic regression models for poor outcome after MT.

NHR	Model 1 OR (95% CI)	*p* value	Model 2 OR (95% CI)	*p* value	Model 3 OR (95% CI)	*p*-value
NHR as a continuous variable						
Per 1-SD increase	1.46 (1.151–1.853)	0.002	1.620 (1.259–2.084)	<0.001	1.445 (1.095–1.908)	0.009
NHR tertile						
Tertile1 (<6.29)	Reference		Reference		Reference	
Tertile2 (6.29–9.50)	1.169 (0.695–1.966)	0.556	1.436 (0.833–2.476)	0.193	1.618 (0.913–2.865)	0.099
Tertile3 (>9.50)	2.371 (1.368–4.112)	0.002	2.887 (1.618–5.151)	<0.001	2.311 (1.248–4.278)	0.008

## Discussion

To our knowledge, this is the first study to evaluate the correlation between NHR and futile recanalization after MT in acute LVO stroke patients. It was found that elevated NHR levels within 24 h post-surgery were significantly associated with adverse prognosis in patients achieving complete recanalization.

Neuroinflammation is associated with BBB disruption, neuronal damage, and worse clinical outcomes following ischemic stroke (18). After the initial cerebral ischemic injury, the inflammatory response exacerbates the damage. Neutrophils are identified as pivotal drivers of ischemic brain injury development (19, 20). Research indicates that neutrophils are the first peripheral blood cells to cross the BBB following ischemic stroke, with their numbers increasing rapidly within hours of stroke onset (21). By releasing reactive oxygen species, proteases (e.g., metalloproteinases, elastase, and cathepsin G), cytokines (e.g., IL-6, IL-8, and TNF-*α*), and chemokines (e.g., CCL2, CCL3, and CCL5), the BBB is compromised, thereby aggravating ischemic damage and cerebral edema (22). Moreover, neutrophil extracellular traps (NETs) formed due to excessive neutrophil activation can trap other blood cells, resulting in pathological thrombosis and amplifying neuroinflammatory responses (23). Angiogenesis and vascular remodeling are essential for post-stroke brain repair (24). Kang et al. found that peptidyl arginine deiminase 4 (PAD4), an enzyme required for NETs formation, is upregulated in peri-ischemic brain tissue. Overexpression of PAD4 exacerbates vascular damage and impedes angiogenesis and vascular repair by releasing additional NETs (25). Several clinical studies have confirmed the potentially detrimental effects of neutrophils on ischemic brain tissue. Cui et al. reported that an increase in neutrophil counts on the second day after the onset of large hemispheric infarction (LHI) was associated with brain herniation and early death (26). Meanwhile, Semerano et al. demonstrated that higher neutrophil counts at admission and on the first day following thrombectomy were significantly associated with poor functional outcomes and increased mortality at 90 days (27).

HDL-C exerts anti-atherosclerotic and anti-inflammatory effects, thereby reducing BBB disruption in patients with acute ischemic stroke (AIS) (28). Atherosclerosis is a common cause of ischemic stroke and is characterized by the accumulation of macrophages and T lymphocytes in the arterial intima (29, 30). Macrophages take up oxidized low-density lipoprotein and transform it into foam cells. In contrast, HDL-C can promote cholesterol efflux from foam cells and exhibits antioxidant and anti-inflammatory properties (31). In addition to its role in reverse cholesterol transport, HDL-C interacts with platelets, the coagulation cascade, and the vascular endothelium. Native HDL-C prevents platelet hyperreactivity by restricting cholesterol overload within platelets and modulating platelet signaling pathways after binding to platelet HDL receptors such as scavenger receptor B and apolipoprotein E receptor 2. Its anti-thrombotic properties are also associated with the inhibition of coagulation cascade reactions and the promotion of clot fibrinolysis. Moreover, HDL-C stimulates endothelial cells to produce nitric oxide and prostacyclin, which are potent inhibitors of platelet activation (32). In a cohort study of Chinese community-based hypertensive patients, higher HDL-C levels were identified as an important protective factor against first-time ischemic stroke (33). Li et al. found that reduced HDL-C levels were independently associated with an increased adverse prognosis at 3 months after AIS and cerebral hemorrhage (34).

Furthermore, HDL-C and neutrophil levels demonstrated reciprocal functional inhibition. Animal experiments conducted by Scanu et al. showed that HDL-C can reduce neutrophil infiltration by inhibiting cytokine synthesis, including IL-6 and IL-1β, thereby alleviating inflammatory responses (35). Apolipoprotein A-I, the primary component of HDL-C (36), decreases neutrophil production by reducing granulocyte colony-stimulating factor levels and restricts neutrophil chemotaxis to localized inflammatory sites by inhibiting IL-8 synthesis in activated neutrophils (37, 38). Previous studies have indicated that activated neutrophils can mediate the oxidation of HDL through oxidant-generating enzymes, such as myeloperoxidase, NADPH oxidase, and nitric oxide synthase. This process impairs the cholesterol efflux capacity and promotes atherosclerosis and inflammatory responses (39, 40). In addition, Carlucci et al. found that low-density granulocytes, a distinct neutrophil subset present in systemic lupus erythematosus, interact with modified HDL to promote foam cell formation and contribute to the progression of inflammatory high-risk plaques (41). Thus, elevated NHR levels may be associated with increased neutrophil count or decreased HDL-C levels. Inflammation and dyslipidemia are key factors influencing the pathophysiology of ischemic stroke (42). As a comprehensive indicator, NHR could better reflect patients’ inflammatory status and lipid metabolism throughout the entire pathological process of stroke.

Our study has several limitations. First, as a retrospective study, the exclusion of participants with missing data may have resulted in the loss of valuable information and introduced some bias into the results. Second, although this was a multicenter study, the sample size was relatively small and did not account for the potential effects of diet, medication, and health status on lipid levels. Therefore, future prospective studies with larger sample sizes and more centers are required to validate our findings. Third, the relationship between NHR and early functional deterioration or related complications (such as symptomatic intracranial hemorrhage, malignant brain edema, post-stroke pneumonia, and urinary tract infection) was not explored. Finally, this study only included NHR within 24 h after MT, and the relatively low AUC of NHR indicated its limited predictive performance. Future studies should explore dynamic changes in NHR and compare its predictive performance with other inflammatory markers to comprehensively evaluate the prognostic value of NHR as an inflammatory predictor.

## Conclusion

Our findings suggest that an elevated NHR is associated with an increased risk of adverse prognosis following complete recanalization after MT in acute LVO stroke patients. NHR could, therefore, serve as a potential inflammatory marker for identifying high-risk patients. Early intervention targeting the inflammatory state may help improve clinical outcomes following thrombectomy.

## Data Availability

The original contributions presented in the study are included in the article/Supplementary material, further inquiries can be directed to the corresponding author.
